# Season, but not symbiont state, drives microbiome structure in the temperate coral *Astrangia poculata*

**DOI:** 10.1186/s40168-017-0329-8

**Published:** 2017-09-15

**Authors:** Koty H. Sharp, Zoe A. Pratte, Allison H. Kerwin, Randi D. Rotjan, Frank J. Stewart

**Affiliations:** 1 Department of Biology, Marine Biology and Environmental Science, Roger Williams University, 1 Old Ferry Road, Bristol, RI 02809 USA; 2 Georgia Institute of Technology, Atlanta, USA; 3 University of Connecticut, Storrs, CT USA; 4 Boston University, Boston, USA; 5 New England Aquarium, Boston, USA

**Keywords:** Coral microbiome, temperate corals, *Astrangia poculata*

## Abstract

**Background:**

Understanding the associations among corals, their photosynthetic zooxanthella symbionts (*Symbiodinium*), and coral-associated prokaryotic microbiomes is critical for predicting the fidelity and strength of coral symbioses in the face of growing environmental threats. Most coral-microbiome associations are beneficial, yet the mechanisms that determine the composition of the coral microbiome remain largely unknown. Here, we characterized microbiome diversity in the temperate, facultatively symbiotic coral *Astrangia poculata* at four seasonal time points near the northernmost limit of the species range. The facultative nature of this system allowed us to test seasonal influence and symbiotic state (*Symbiodinium* density in the coral) on microbiome community composition.

**Results:**

Change in season had a strong effect on *A. poculata* microbiome composition. The seasonal shift was greatest upon the winter to spring transition, during which time *A. poculata* microbiome composition became more similar among host individuals. Within each of the four seasons, microbiome composition differed significantly from that of surrounding seawater but was surprisingly uniform between symbiotic and aposymbiotic corals, even in summer, when differences in *Symbiodinium* density between brown and white colonies are the highest, indicating that the observed seasonal shifts are not likely due to fluctuations in *Symbiodinium* density.

**Conclusions:**

Our results suggest that symbiotic state may not be a primary driver of coral microbial community organization in *A. poculata*, which is a surprise given the long-held assumption that excess photosynthate is of importance to coral-associated microbes. Rather, other environmental or host factors, in this case, seasonal changes in host physiology associated with winter quiescence, may drive microbiome diversity. Additional studies of *A. poculata* and other facultatively symbiotic corals will provide important comparisons to studies of reef-building tropical corals and therefore help to identify basic principles of coral microbiome assembly, as well as functional relationships among holobiont members.

**Electronic supplementary material:**

The online version of this article (10.1186/s40168-017-0329-8) contains supplementary material, which is available to authorized users.

## Background

Corals are the foundation of tropical reef ecosystems worldwide and are increasingly known to harbor prevalent and ecologically important microbial communities (reviewed in [1]). The now classic two-partner symbiosis between the coral host and intracellular photosynthetic symbiont (*Symbiodinium*) has been updated to include the microbiome, with corals now regarded as meta-organisms [2]: metazoan hosts with diverse bacterial, archaeal, fungal, protistan, and viral partners [3]. Much of the coral microbiome research over the past two decades has focused on prokaryotic communities and has consistently demonstrated that prokaryotes in tropical corals play a critical role in nutrient acquisition and waste processing [4, 5] and host resistance to pathogens [6, 7] and are therefore largely beneficial to corals and coral reefs [3, 8, 9]. Most corals appear to acquire their specific bacterial component from seawater during early life stages [10, 11], although intergenerational transmission may also occur [12, 13]. Yet, coral-associated microbiomes are distinct from those in the surrounding seawater and sediment [14–17], and the mechanisms that regulate the composition of the coral microbiome remain largely unknown. These could include variation in the host’s physiology (e.g., due to developmental stage), the activity and abundance of host-associated *Symbiodinium*, and the chemical and physical properties of the surrounding environment, with all of these potentially linked to both natural (seasonal) and anthropogenic changes in ocean conditions.

Research suggests that the native coral microbiome changes in the face of environmental stress [1, 7, 8, 18–21]. Tropical coral reefs are threatened by a wide variety of stressors, including pollution, overfishing, ocean acidification, and ocean warming, with the latter capable of affecting corals at the global scale [22–24]. Notably, recent sustained warming events [25] have triggered massive coral bleaching across ocean basins. Bleaching, which involves loss of intracellular photosynthetic *Symbiodinium*, triggers rapid changes in physiological interactions in tissue, skeleton, and mucus layers, with observed taxonomic and genomic changes in the coral microbiome [19, 26, 27]. In tropical corals, sustained warming events and resultant loss of *Symbiodinium* (bleaching) have been shown to result in a decrease in defensive properties of coral mucus and bioactivity against known coral pathogens [7], potentially allowing for infection and disease [9, 28]. However, the biochemical processes that drive these microbiome shifts are still not well understood, and it is unclear whether the shifts that follow bleaching are a response to the loss of *Symbiodinium*, to increased temperature, or to both. It is therefore crucial to assemble a model of microbe-microbe interactions within the coral meta-organism that can describe the relative roles of *Symbiodinium* versus environmental conditions, such as temperature, as drivers of prokaryotic diversity.

**Fig. 1:**
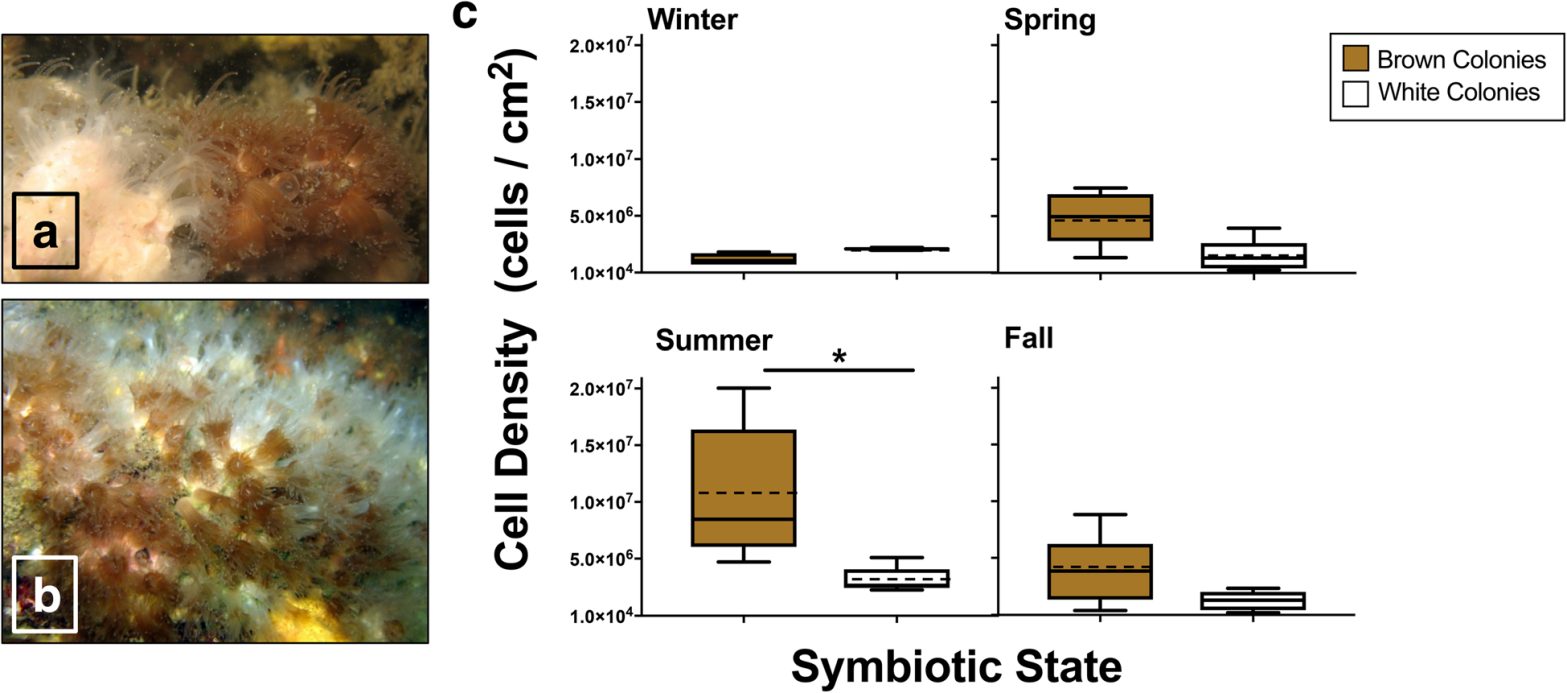
Underwater photographs of (**a**) an aposymbiotic *A. poculata* colony directly adjacent to a symbiotic *A. poculata* colony and (**b**) a mixed *A. poculata* colony. Photos: J. Dimond. (**c**) Median *Symbiodinium psygmophilum* density (# *Symbiodinium* cells·cm^−2^ coral) of colonies, with brown and white designations based on visual assessment in the field. Boxplots represent the 25th to 75th percentile range of *Symbiodinium* density among sample replicates for each season, while whiskers indicate the full range of sample values. Two-way ANOVA shows that both collection season and colony type affected *S. psygmophilum* density (see Additional file 1), while post hoc Tukey tests found that summer brown colonies had a higher *S. psygmophilum* density compared to those from any other season or colony type (asterisk). Mean indicated by dotted line where different from median

Studies of corals with naturally varying *Symbiodinium* levels, notably if spanning seasonal fluctuations in temperature, provide valuable opportunities to tease apart interactions among environmental conditions, *Symbiodinium*, and prokaryotic communities in corals. Because most tropical corals have an obligate relationship with *Symbiodinium*, it is impossible to distinguish stress due to symbiont loss from stress due to the environmental change that resulted in symbiont loss. The facultatively symbiotic temperate coral *Astrangia poculata* (the “Northern Star Coral”; Fig. 1) is an ideal alternative study system for exploring the interplay among members of the coral meta-organism without unnaturally disturbing the typically obligate symbiosis. *A. poculata* engages in facultative symbiosis with only one species of symbiont, *Symbiodinium psygmophilum* (ITS2 Type B2) [29, 30]. Sympatric *A. poculata* individuals can exhibit wide variation in *S. psygmophilum* density [31]. As a temperate species, *A. poculata* also experiences a wide range of temperatures throughout the year. In the species’ northernmost range (southern New England), seawater temperature fluctuates over a range exceeding 20 °C, with average temperatures from 4 to 29 °C across seasons (NOAA Tides & Currents, Newport, RI: site 8452660). As a consequence, *A. poculata* metabolic rates vary seasonally, with the coral entering into quiescence (dormancy) during winter months [30, 32, 33], with reduced symbiosis during this quiescent period [34]. Studies of *A. poculata* in its natural environment across seasons therefore allow testing of the influence of symbiosis and seasonality on other aspects of the meta-organism.

Here, we present the first description of prokaryotic diversity in the coral *A. poculata,* sampled over seasons from a location in Narragansett Bay, Rhode Island, near the northernmost limit of the species range. In this study, we refer to the associated prokaryotic community as the “microbiome.” The sampled *A. poculata* individuals vary in symbiotic state, or density of *S. psygmophilum* cells in coral tissue, as (a) symbiotic or “brown”; (b) aposymbiotic or “white”; or (c) “mixed” (colonies are mottled and have localized regions of relatively high cell density). Symbiotic states have previously been assigned according to color and approximated chlorophyll concentration [32, 35, 36] and cell density of *Symbiodinium* in summer months [37], with brown colony *Symbiodinium* densities exceeding 10^6^ cells cm^−2^ coral tissue, and white colony *Symbiodinium* densities ranging from 10^4^ to 10^6^ cells cm^−2^ [37].

By sampling both across seasons and across colonies varying in symbiotic state, our objective is to determine whether the taxonomic structure of the microbiome in *A. poculata* is linked to symbiotic state or to other factors that fluctuate seasonally. We hypothesized that (1) microbiome composition would vary significantly across seasons and (2) within seasons, especially within summer, symbiotic state would be a significant driver of microbiome composition.

## Methods

### Coral and seawater specimen collection

Whole colonies of symbiotic (brown) and aposymbiotic (white) *A. poculata* were collected via SCUBA at Fort Wetherill State Park, Jamestown, RI (41° 28′ 40″ N, 71° 21′ 34″ W) in fall (September 9, 2015), winter (March 5, 2016), spring (April 29, 2016), and summer (July 19, 2016). To minimize the potential of hyper-localized environmental effects due to benthic composition, benthic orientation, or abiotic factors, we sampled colony pairs in which a brown colony occurred within 10 cm of a white colony. At each time point, colony pairs ranging in size from 4 to 8 cm total diameter were collected from ~1 to 5 m water depth from the same location (*n* = 10 colony pairs in each of fall, spring, and summer; *n* = 6 colony pairs in winter). Immediately post-collection, subsamples of each colony were frozen in liquid nitrogen at the surface and stored at −80 °C until DNA extraction. The remainder of each colony was transported in individual, labeled Whirl-Pak® bags within a cooler of seawater back to the laboratory for immediate (within 2–3 h of collection) *Symbiodinium* quantification. During each collection, replicate seawater samples (*n* = 10 for winter, spring, summer; *n* = 9 for fall, summarized in Table 1) were collected from ~1 to 5 m water depth using autoclaved glass 1 l bottles. Immediately after water collection, seawater microbes were filtered by syringe onto a Sterivex™ cartridge (0.2-μm pore size), which was then frozen in liquid nitrogen and stored at −80 °C until DNA extraction. Daily measurements of seawater surface temperatures (NOAA 172 Tides & Currents, Newport, RI: site 8452660) spanning the year-long sampling period were used to assign temperature ranges to each season. Average sea surface temperatures in the four sampling periods were 22 °C in fall, 3 °C in winter, 9 °C in spring, and 21 °C in summer (NOAA 172 Tides & Currents, Newport, RI: site 8452660).

**Table 1:** Summary of collected specimens via SCUBA at Fort Wetherill State Park, Jamestown, RI (41° 28′40″ N, 71° 21′34″ W) in Fall (9/9/15), Winter (3/5/16), Spring (4/29/16) and Summer (7/19/16) time points. Shown are the number of replicate specimens collected for each sample group during each time point. The total number of high-quality reads, including the high-quality reads from all seawater and all coral specimens, is shown

	Number of samples (total = 110)				Total reads	OTUs
	Fall	Winter	Spring	Summer		
Seawater	9	9	10	10	7,683,584	37,089
Brown colonies	10	6	10	10		28,243
White colonies	10	6	10	10		

### *Symbiodinium* quantification

The relationship between polyp color and areal chlorophyll concentration has been previously established for *A. poculata* [32]. Here, we visually distinguished brown from white corals during collection and subsequently measured *Symbiodinium* density from each colony via cell counts (# *Symbiodinium* cells cm^−2^ coral). From each colony, coral tissue was removed from the skeleton using a Waterpik [38] with 0.22-μm filtered seawater, homogenized, and centrifuged to produce a pellet. Excess supernatant was removed, and pellets were suspended in a final volume of 12–15 ml. Samples were fixed in 4% zinc-formalin fixative Z-Fix (Anatech, Ltd., Battle Creek, MI) and stored at room temperature. Subsampled colony surface area was determined using aluminum foil as in Marsh [39]. Average total number of *Symbiodinium* cells was enumerated for each waterpiked subsample (above) using a hemocytometer (*n* = 4 counts per sample replicate), with a total of five replicate subsamples counted per colony. Because the collected corals were small and the subsamples were prioritized for DNA extraction, not all colonies were used for symbiont quantification. Photos of each colony were taken with a color standard as previously described [32, 36] and were used for visual confirmation of color and symbiotic state assignment. A two-way ANOVA was used to determine the effect of collection season and colony symbiotic state (“brown” or “white”) on *Symbiodinium* density, followed by post hoc Tukey tests.

To quantify symbiotic state across depth in the field, underwater transects were conducted in Ft. Wetherill, RI, in July 2010. Two divers were deployed along a vertical granite slope to conduct a 15-m transect, simultaneously surveying and marking colonies as either “brown,” “white,” or “mixed.” Every 0.6 m along a linear transact, *A. poculata* colonies were counted in a 0.25 m^2^ quadrat, centered around the transect line. All *A. poculata* colonies inside the quadrat were included.

### DNA processing

From each *A. poculata* subsample, total DNA was extracted from a small fragment (~1 cm^2^) that included coral tissue, mucus, and skeleton, using the PowerSoil® DNA Isolation Kit (Qiagen, Valencia, CA). Each fragment (only partially thawed) was placed directly into a PowerBead tube for extraction according to the manufacturer’s protocol. DNA from seawater microbes was extracted directly from the Sterivex™ cartridges using a phenol:chloroform extraction method (modified from [16, 40]). Following the phenol:chloroform extraction, a second extraction was performed using the PowerSoil® DNA Isolation Kit. The V4 region of the 16S rRNA gene was amplified for all samples using F515 and R806 primers [41], with the addition of Illumina-specific adapters [42]. Final PCR reactions consisted of 22 μl Platinum PCR SuperMix (ThermoFisher Scientific, Waltham, MA), 2 μl DNA template, 0.5 μl each of the primers F515 (5′-CACGGTCGKCGGCGCCATT-3′) and R806 (5′-GGACTACHVGGGTWTCTAAT-3′) (final concentrations 0.4 μM each), and 0.5 μl BSA (20 mg/ml, New England BioLabs, Inc., Ipswich, MA). Amplicons were generated with an initial denaturation of 3 min at 94 °C, followed by 30 cycles of 94 °C (45 s), 55 °C (45 s), and 72 °C (90 s), with a final extension for 10 min at 72 °C, and cleaned using Diffinity RapidTips (Chiral Technologies, Inc., West Chester, PA). All PCR products were pooled in equimolar concentration and sequenced on the Illumina MiSeq (2 × 250 bp paired end) with a 10% PhiX control. It is important to recognize that the primers used in the study may not have captured the full extent of microbial diversity, particularly within the Archaea. It is therefore possible that patterns of community assembly may differ among certain subsets of the microbiome if evaluated at higher levels of resolution.

### Sequence data analysis

Only sequences greater than 100 bp with a Phred score greater than 25 were retained, as determined by Trim Galore!. Paired end reads were then merged using FLASH [43], imported into QIIME version 1.9.1 [44], and checked for chimeric sequences using USEARCH 6.1 against the Greengenes database release 8.15.13 [45]. After removal of chimeric sequences, taxonomy was assigned using an open-reference algorithm [46] against the Greengenes database. Reads that were not identified as “Archaeal” or “Bacterial” were removed, as well as reads that identified as “Chloroplast.” An OTU table rarefied to 1000 reads was used for all analyses, with the exception of DESeq2 analysis [47] conducted through the R package Phyloseq [48], which contains an internal normalization algorithm.

### Taxonomic diversity analysis

Alpha diversity was analyzed in QIIME at a uniform sequence depth (*N* = 1000) using the Shannon Index (H′), Shannon Equitability (E_H_), Chao1, and phylogenetic diversity (PD) metrics. The Shannon Index (H′) was converted to a natural log Shannon Index. Alpha diversity data were then analyzed in GraphPad Prism v6.0h for Mac OS X. A two-way ANOVA was used to determine the effect of collection season and colony symbiotic state (“brown” or “white”) on coral microbial alpha diversity, and a one-way ANOVA was used to analyze the effect of collection season on seawater microbial alpha diversity. Similarity in community composition was tested via ANOSIM and PERMANOVA in QIIME using 999 permutations. Variation in community composition (beta diversity) among samples was visualized via non-metric multidimensional scaling (NMDS) ordination based on Bray–Curtis dissimilarity matrices, using the VEGAN package [49].

## Results

### *Symbiodinium psygmophilum* densities and facultative symbiosis in *A. poculata*

Cell counts were used to measure *Symbiodinium* cell density from colonies that were visually assessed as “brown” or “white” corals during collection. Two-way ANOVA demonstrated that both season and colony color were correlated with *Symbiodinium* density (Additional file 1). However, the magnitude of seasonal variation differed between brown and white corals. Post hoc Tukey tests demonstrated that *Symbiodinium* density did not differ across seasons in white colonies. In contrast, brown colonies exhibited significantly higher *Symbiodinium* density in summer compared to other seasons, which did not differ from one another (Fig. 1c). Notably, *Symbiodinium* density in brown colonies increased an order of magnitude from winter (1.8 × 10^6^ cells/cm^2^) to summer (1.9 × 10^7^ cells/cm^2^; Fig. 1c). During summer, brown colonies also exhibited significantly higher *Symbiodinium* density compared to white colonies. Adjacent *A. poculata* colonies displayed different symbiotic states even at the same depth within a site (Additional file 2).

### *Astrangia poculata* and seawater microbiome sequence statistics

Small subunit ribosomal RNA (16S rRNA) gene amplicons from a total of 110 coral and seawater specimens were sequenced (summarized in Table 1). At each seasonal time point, whole coral colony material (skeleton, tissue, and mucus together) was collected from both brown and white colonies, and seawater samples were collected. Sequencing of these samples produced 7,683,584 high-quality sequence reads, which were clustered into operational taxonomic units (OTUs) based on 97% sequence similarity. Fifty thousand seven hundred fifty of the total 51,213 OTUs were affiliated with members of the domain Bacteria, and 463 of the OTUs were affiliated with the domain Archaea. Coral and seawater specimens harbored a total of 28,243 and 37,089 OTUs, respectively.

### Influence of seasonality and seawater on *A. poculata* microbiome diversity

**Fig. 2:**
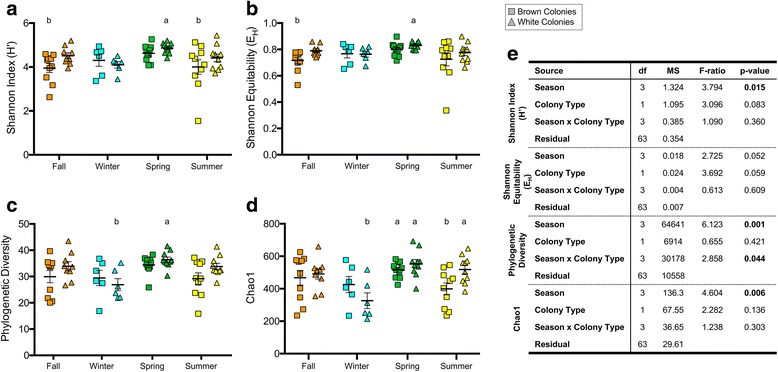
Alpha diversity metrics of brown and white colonies. All indices are highest for white colonies collected during the spring (**a**–**d**). Two-way ANOVA demonstrated that collection season but not colony type affected coral microbial alpha diversity (**e**). Bolded *p* values were significant (*p* < 0.05). Results from a post hoc Tukey test are presented as letters (**a**, **b**) denoting groups that are significantly different. Columns that lack letters are not significantly different

Two-way ANOVA analysis of four indices of alpha diversity, including Chao1, Shannon Diversity (H′) and Equitability (E_H_), and phylogenetic diversity (PD), demonstrated a significant influence of season on coral microbial alpha diversity (Fig. 2, two-way ANOVA; H′ *p* = 0.015; E_H_ *p* = 0.052; PD *p* = 0.001; Chao1 *p* = 0.006), with both H′ and E_H_ metrics based on a combination of richness and evenness, PD expressing overall phylogenetic diversity in the community, and Chao1 estimating richness by taking into account the number of rare OTUs present in the community. Post hoc Tukey tests revealed that spring white *A. poculata* colonies exhibited significantly increased H′, E_H_, and PD compared to all other sample types. The influence of rare OTUs (Chao1) in spring white colonies was similar to that in summer white and spring brown colonies. In contrast, the microbiomes of winter white colonies had lower levels of phylogenetic diversity and a smaller number of rare OTUs compared to almost all other sample types. Fall and summer brown colonies were significantly lower in richness and evenness (H′) compared to other sample types, while summer brown colonies also had a relatively smaller number of rare OTUs compared to those of most other sample types (with the exception of winter whites, Fig. 2).

**Fig. 3:**
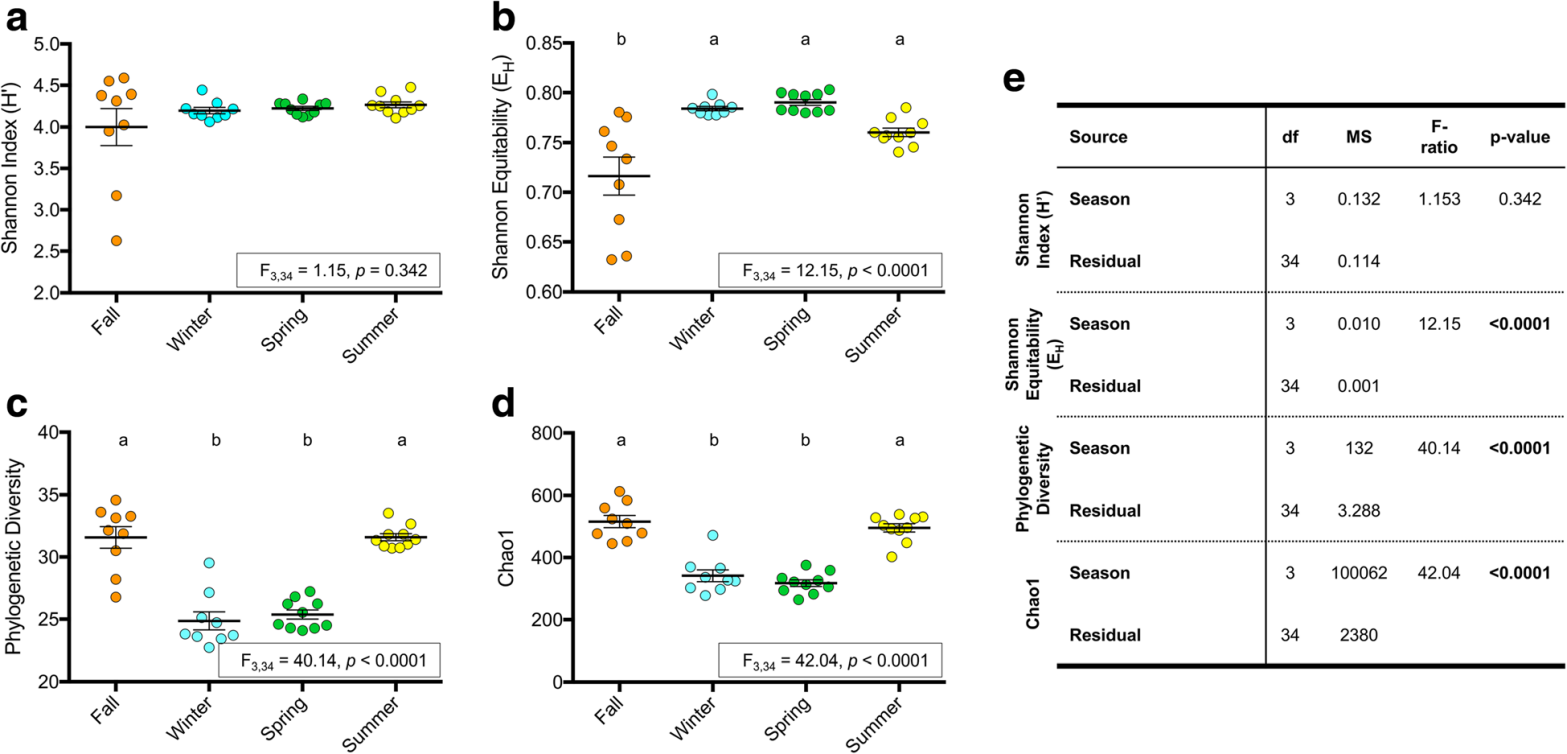
Alpha diversity metrics in seawater samples. The values suggest varying patterns of microbial diversity in the seawater (**a**–**d**). One-way ANOVA demonstrated that season consistently affected seawater alpha diversity (**e**). Bolded *p* values were significant (*p* < 0.05). Results from a post hoc Tukey test are presented as letters (**a**, **b**) denoting groups that are significantly different. Columns that lack letters are not significantly different

One-way ANOVA analysis of multiple indices of alpha diversity demonstrated that season significantly impacts seawater microbial alpha diversity (Fig. 3, one-way ANOVA; H′ *p* = 0.342, E_H_/PD/Chao1 *p* < 0.0001). However, the seasonal effect varied depending on the metric and did not parallel the trend observed in coral. While seawater H′ values did not vary among seasons, E_H_ values (equitability) of winter, spring, and summer seawater communities were similar and significantly higher than those in fall (post hoc Tukey tests, Fig. 3). In contrast, seawater PD and Chao1 values exhibited the opposite pattern, being significantly higher in summer and fall compared to winter and spring. Thus, compared to winter and spring communities, summer and fall seawater microbiomes were less even, with total richness affected less by rare taxa, potentially consistent with dominance by a subset of active community members.

**Fig. 4:**
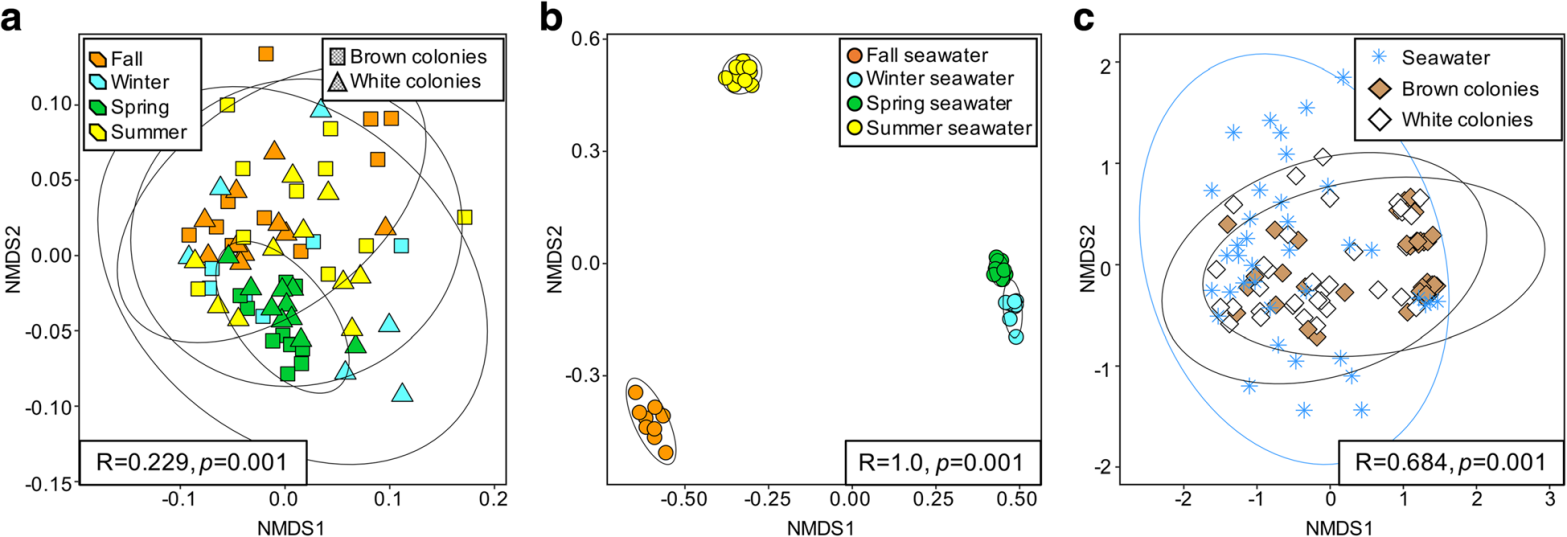
NMDS visualizations of beta diversity analysis using the Bray–Curtis metric. Ellipses represent 95% confidence intervals. ANOSIM (boxes within NMDS plots) also revealed significant dissimilarity between groupings of coral samples from the four seasonal time points (**a**), of seawater samples from the four seasonal time points (**b**), and of brown colonies, white colonies, and seawater (**c**)

**Fig. 5:**
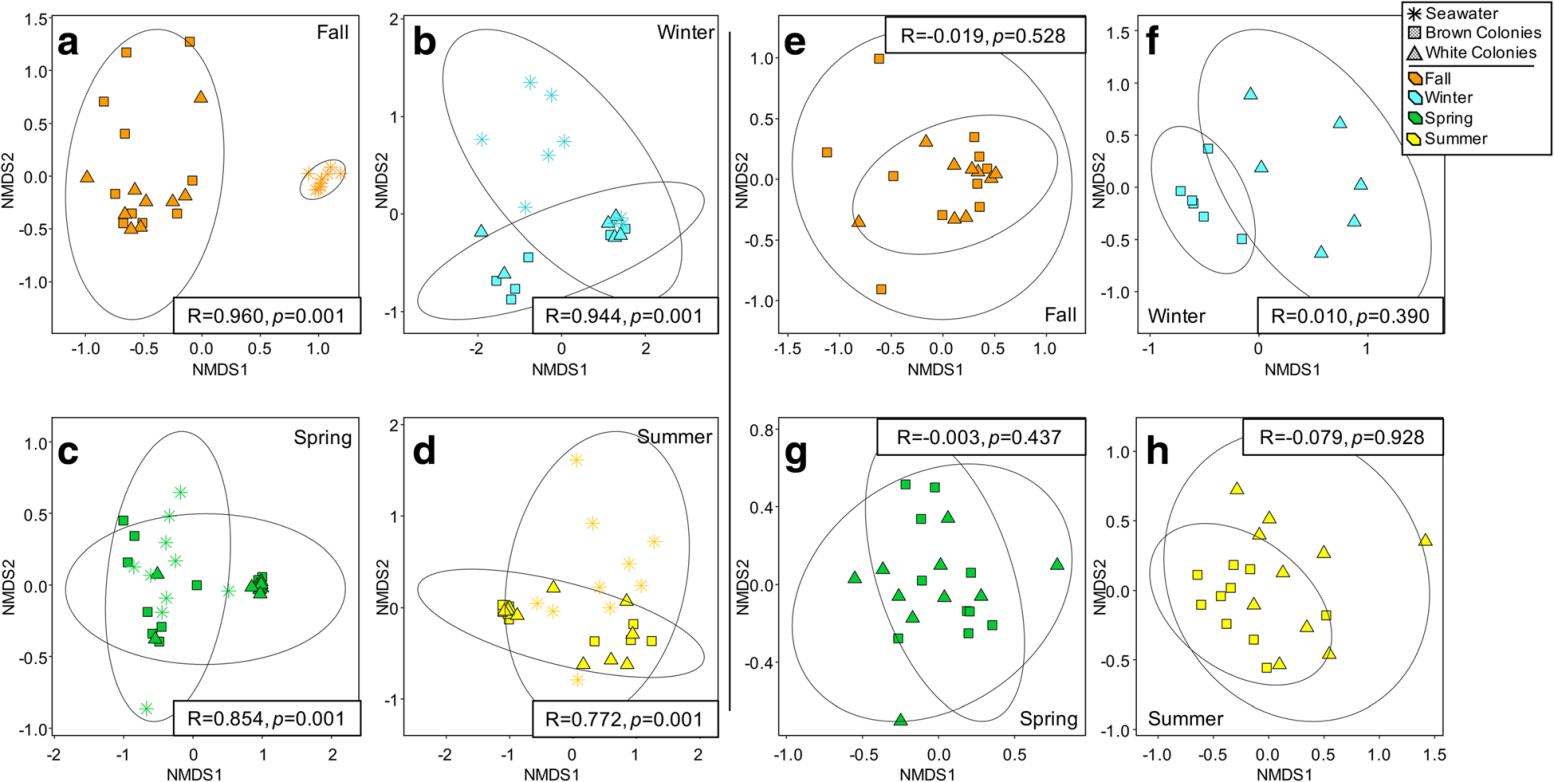
NMDS visualizations of beta diversity analysis using the Bray–Curtis metric separating samples by collection season. Ellipses represent 95% confidence intervals for coral versus seawater samples (**a**–**d**) or for brown versus white colonies (**e**–**h**). In each seasonal time point, ANOSIM (boxes within NMDS plots) did not reveal significant dissimilarity between symbiotic state of colonies but did reveal significant dissimilarity between corals and seawater

Both *A. poculata* microbiome composition (Fig. 4a, ANOSIM *R* = 0.229, *p* = 0.001, PERMANOVA, *F* = 2.91, *p* = 0.001) and seawater microbiome composition (Fig. 4b, ANOSIM *R* = 1.0, *p* = 0.001, PERMANOVA, *F* = 78.01, *p* = 0.001) varied significantly across seasonal time points. This variation was notably higher in the seawater, with seawater samples partitioning into non-overlapping NMDS clusters based on season (Fig. 4a, b). Both brown and white *A. poculata* microbiomes differed significantly from seawater microbiomes when the data from all seasons were grouped (Fig. 4c; ANOSIM *R* = 0.684, *p* = 0.001, PERMANOVA, *F* = 18.51, *p* = 0.001) and when evaluated independently by season (Fig. 5a–d; ANOSIM *R* = 0.772–0.960, *p* = 0.001, PERMANOVA, *F* = 14.62–23.09, *p* = 0.001), suggesting minimal effects of seawater microbiomes on coral microbiome composition. While the coral microbiome substantially overlaps with that of the seawater microbiome, it appears to be comparatively restricted (lower dispersion, Fig. 4c). However, the level of relatedness between coral and seawater microbiomes varied among seasons, with coral and seawater communities being least similar in the fall (ANOSIM *R* = 0.960, *p* = 0.001, PERMANOVA, *F* = 17.48, *p* = 0.001) and most similar in summer (ANOSIM *R* = 0.772, *p* = 0.001, PERMANOVA, *F* = 14.62, *p* = 0.001, Fig. 5a–d). Conversely, the spring coral and seawater microbiomes were found to be the least similar by the PERMANOVA metric (*F* = 23.09, *p* = 0.001, ANOSIM *R* = 0.854, *p* = 0.001), likely due to the lower dispersion of the spring coral samples, to which PERMANOVA is more sensitive.

Springtime coral microbiomes were distinct from those in other seasons. Removal of the spring data revealed only slight differences in coral microbiome composition among the other three seasons (Additional file 3, ANOSIM *R* = 0.135, *p* = 0.002, PERMANOVA *F* = 2.04, *p* = 0.001). Furthermore, although non-overlapping season-specific clusters were not observed in NMDS analysis of the *A. poculata* microbiomes (Fig. 4a), those from the spring were most similar to one another, exhibiting the lowest inter-sample variability (dispersion) compared to other seasons. Together, these patterns indicate a relative uniformity of coral microbiomes during spring and that a shift in coral microbiome composition during this period contributed substantially to the overall seasonal variation.

### Influence of symbiotic state on *A. poculata* microbiome composition

In contrast to results based on season, and to our expectations, two-way ANOVA analysis of four indices of alpha diversity did not demonstrate a strong influence of symbiont state (brown or white) on *A. poculata* microbiomes (Fig. 2). A significant difference in alpha diversity between brown and white colonies was observed only for the Chao1 metric (Fig. 2d) and only during summer when *Symbiodinium* density differed the most between brown and white colonies (Fig. 1c). For all other metrics and seasons, alpha diversity of *A. poculata* microbiomes did not differ between brown and white colonies (Fig. 2, two-way ANOVA; H′ *p* = 0.083; E_H_ *p* = 0.059; PD *p* = 0.421; Chao1 *p* = 0.136). Furthermore, symbiotic state had no perceivable effect on taxonomic composition of the microbiome. For each of the four time points, ANOSIM detected no dissimilarity in microbiome composition between brown and white *A. poculata* individuals from the same time point (Fig. 5e–h, for all, ANOSIM *R* < 0.01, *p* > 0.3, PERMANOVA, *F* < 1.06, *p* > 0.34). When season was removed from the analysis, no dissimilarity was found between all brown and all white *A. poculata* individuals (ANOSIM *R* = − 0.014, *p* = 0.844, PERMANOVA, *F* = 0.83, *p* = 0.746).

### Dominant microbiome taxa

**Fig. 6:**
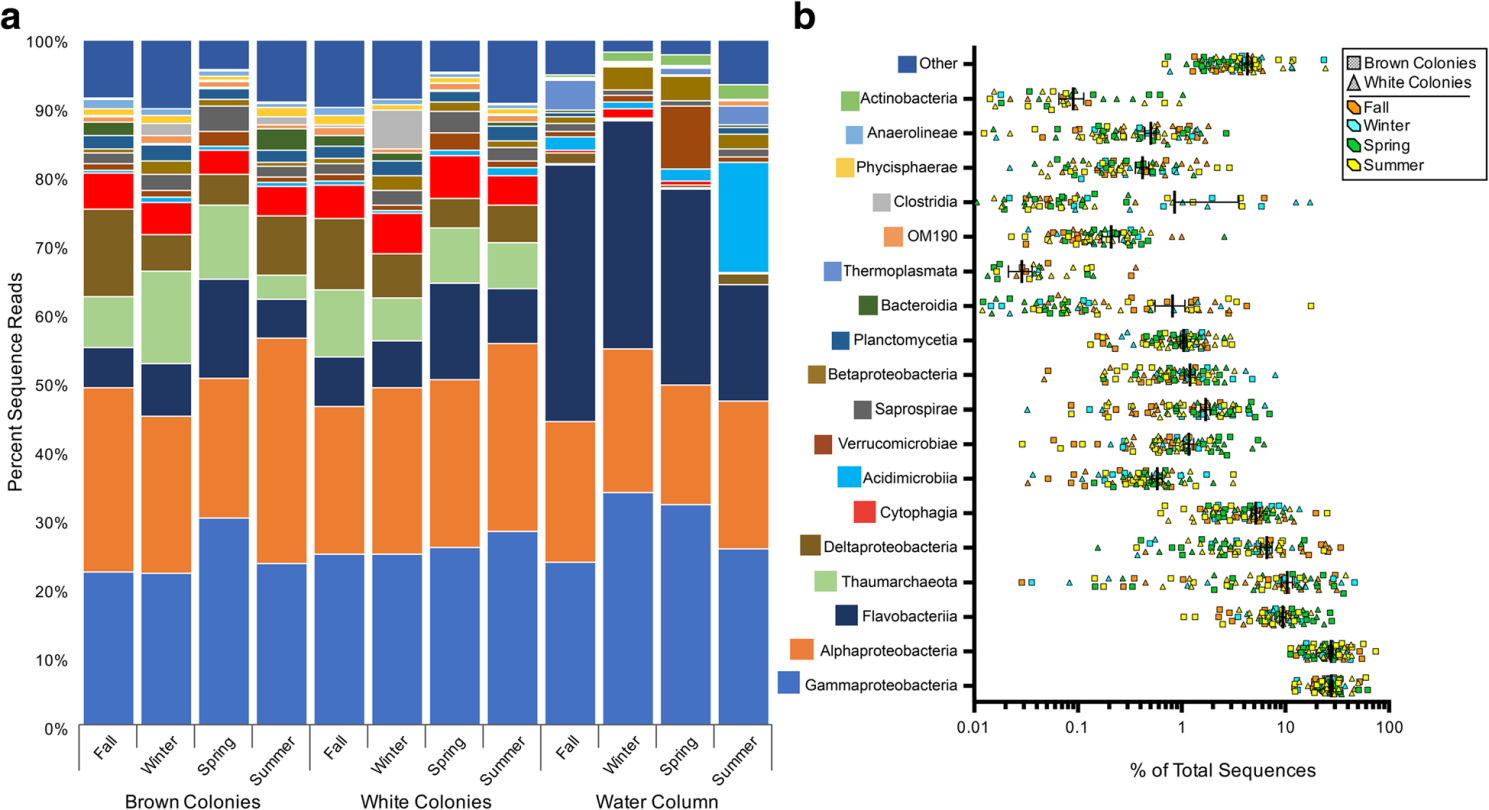
Taxonomic composition of the *A. poculata* and seawater microbiomes at the prokaryotic class level, according to 16S sequence analysis. (**a**) Coral microbiomes are dominated by six prokaryotic classes, and the seawater microbiomes are dominated by three of the same classes, exhibiting more seasonal variation. Bars represent mean abundance (*n* = 10 *A. poculata* colonies in fall, spring, summer; *n* = 6 colonies in winter; *n* = 10 seawater samples in all four time points) of each class. (**b**) Variation in relative abundance of each prokaryotic class in the samples. Relative abundance of each prokaryotic class in the coral microbiomes vary by sample, but not by season or colony type. Taxa present at greater than 1% of the average community are shown. Mean % of total sequences is represented by thick bar, and standard error is represented by thin bars. Scatter plot presented on a log scale to cover range of variation across all classes

**Fig. 7:**
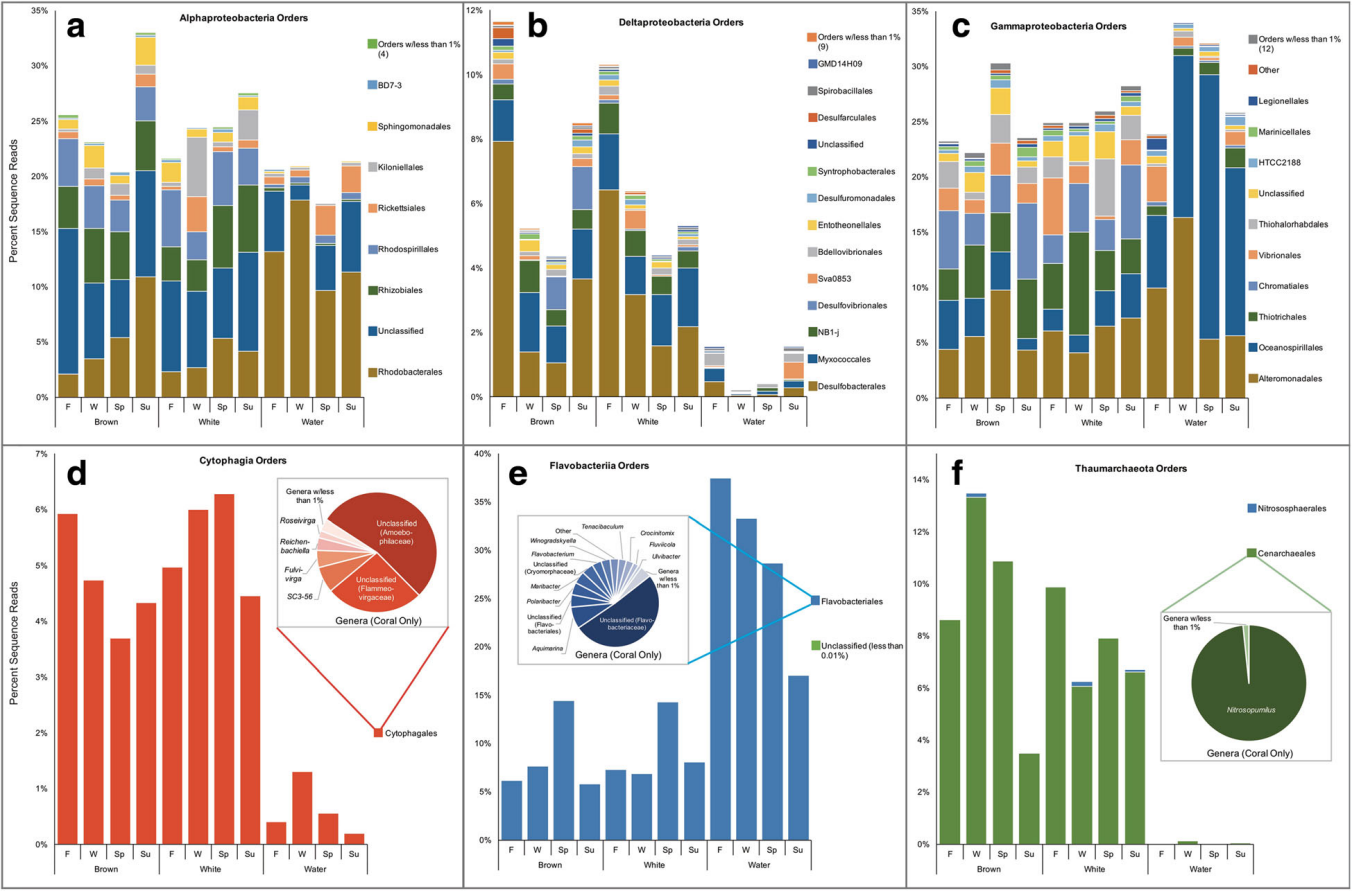
Taxonomic composition of the six most abundant prokaryotic classes from all sample types. Bars represent mean abundance (*n* = 10 for *A. poculata* colonies in fall, spring, summer; *n* = 6 colonies in winter; *n* = 10 for seawater in all four time points). Taxonomic resolution for each class is further broken down to order (**a**–**c**) or genus (**d**–**f**) level

OTUs from this *A. poculata* specimen collection, spanning the four seasonal time points and regardless of symbiotic state, consistently were dominated by six main classes or phyla of prokaryotes: the γ-proteobacteria, α-proteobacteria, δ-proteobacteria, Flavobacteriia, Cytophagia, and Thaumarchaea (Fig. 6). In the seawater samples, three main classes (γ-proteobacteria, α-proteobacteria, and Flavobacteriia) were consistently the most abundant. More γ-proteobacterial orders were present in the *A. poculata* samples than in the seawater (Fig. 7). Notably, in all seasons, the γ-proteobacteria from the corals and the seawater consisted primarily of Alteromonadales and Oceanospirillales, both of which have been implicated in dimethylsulfoniopropionate (DMSP) and polysaccharide degradation in other coral species [50, 51]. Other abundant OTUs in the coral samples included those affiliated with the Chromatiales, Vibrionales, and Thiohalorhabdales. The coral samples also possessed higher order-level richness within the α-proteobacteria, compared to those in the seawater (Fig. 7). The majority of the α-proteobacterial sequences from both coral and seawater samples were consistently Rhodobacterales and an unclassified α-proteobacterium (10% on average in corals; 3–10% in seawater). OTUs representing Rhizobiales, Rhodospirillales, and Sphingomonadales, which made up more than 5% of the OTUs from the coral samples, were detected in the seawater samples, but they made up less than 1% of the OTUs from the seawater. The coral samples consistently had a higher proportion of OTUs representing δ-proteobacteria (2–11% of OTUs) compared to the seawater samples (less than 2% of OTUs). These OTUs were primarily Desulfobacterales and Myxococcales (Fig. 7). Other δ-proteobacterial orders consistently present in the corals included Entotheonellales, which has been observed previously in a diversity of marine sponges [52, 53], and the candidate division NB1-*j*, documented previously in tropical hard corals [54]. A single OTU representing the Thaumarchaeota genus *Nitrosopumilus*, which never contributed to more than 0.2% of the sequences in the seawater samples, was detected in 100% of *A. poculata* samples and averaged 8% of the sequences (1%–36% of the sequences in a given sample). This Thaumarchaeota OTU did not vary significantly in relative abundance (DESeq2 analysis) according to season or *A. poculata* symbiotic state (see Additional file 4). Cytophagia sequences recovered from the corals similarly represented few genera, and a majority fell within the genus *Amoebophilus* (Fig. 7), previously proposed as a symbiont of *Symbiodinium* within the tropical coral *Pocillopora meandrina* [55]. Flavobacteriia sequences exhibited lower relative abundance in corals (5–14% of total sequences) compared to seawater (17–36%, Fig. 7). Flavobacteriia sequences, 50% of which belonged to the same genus (unclassified), spiked in abundance during the spring time point, both in brown and white symbiotic states. Of approximately 28,000 unique OTUs recovered from *A. poculata* samples, 97 were present in ≥ 90% of samples (see Additional file 5), while only 25 OTUs were present across all *A. poculata* samples.

### Microbiome taxa differing in relative abundance by season

The seasonal shifts in the composition of *A. poculata* and seawater microbiomes involved changes in the relative abundance of specific taxa. DESeq2 analysis identified 181 genera whose relative abundance changed significantly in at least one pairwise comparison between seasonal datasets. Compared to *A. poculata* microbiomes from the winter time point, those of the spring time point were significantly enriched in several taxa commonly affiliated with corals (see Additional file 4), including Oceanospirillaceae, Flavobacteriaceae, Microbacteriaceae, Rhodobacteraceae, Bdellovibrionaceae, Alteromonadaceae, and Verrucomicrobiaceae. Notably, one of the Oceanospirillaceae OTUs enriched in the spring belongs to the genus *Portiera*, a genus first described as an endosymbiont in whiteflies [56] and since detected in coral mucus samples in the Florida Reef Tract [57]. The enrichment of Verrucomicrobiaceae sequences from corals in the spring time point is consistent with a seasonal increase in Verrucomicrobiaceae in the seawater samples (Fig. 6). However, a clear connection to the seawater community was not evident for other taxa enriched in springtime corals. For example, members of the Flavobacteriales, though enriched in *A. poculata* in the spring, did not vary in relative abundance in seawater across seasons. The DESeq2 analysis indicated that Clostridiales, Burkholderiaceae, and Xenococcaceae were enriched in the wintertime relative to other seasons. In addition, from winter to spring, OTUs from several groups, including the Vibrionaceae, Alteromonadaceae, and Rickettsiaceae decreased in relative abundance (see Additional file 4). Like Vibrionaceae and Alteromonadaceae, Rickettsiaceae is a bacterial family containing functionally diverse organisms, including several described pathogens. However, this group has also been detected widely in putatively healthy tropical corals [58, 59]. The *A. poculata* microbiome exhibited significant relative increases in members of the Bacteroidales, Clostridiales, and Synechococcus from spring to summer time points, whereas the relative abundance of Rhodobacteraceae, Rickettsiaceae, and Verrucomicrobiaceae decreased significantly in the summer (see Additional file 4).

## Discussion

This study sampled a temperate, naturally facultative symbiotic coral (*Astrangia poculata*) across seasons to test whether symbiotic state (density of the symbiont *Symbiodinium psygmophilum* in the coral tissue) structures the coral-associated microbiome. Determining how the presence, concentration, and activity of *Symbiodinium* affects microbiomes in tropical corals has been challenging because the loss of symbionts (bleaching) constitutes a significant stress to most tropical corals, often resulting in coral death, thereby preventing experimental manipulation of symbiotic state. To our knowledge, our study is the first to decouple coral-*Symbiodinium* symbiosis from seasonal variation to explore the microbiome response in situ. Both brown and white colonies of *A. poculata* harbor microbiomes with taxonomic diversity and composition distinct from that of the surrounding seawater (Fig. 4c). Although we observed differences in alpha diversity between brown and white colonies at some time points, notably in the summer (Fig. 2), within each of the four seasons, microbiome composition was comparatively uniform between symbiotic and aposymbiotic corals (Fig. 4). This was true even in the summer when *Symbiodinum* density was significantly higher in brown colonies. In contrast, microbiome composition exhibited significant shifts across seasons, due mostly to differences associated with the spring time point (Additional file 3, Fig. 4a). Thus, factors that vary with season, other than symbiotic state, are primary determinants of microbiome structure in *A. poculata.*

### Symbiotic state versus other seasonal effects

The finding that symbiotic state has a relatively minor effect on cnidarian microbiome structure is inconsistent with prior studies. Experiments involving the facultatively symbiotic anemone *Aiptasia* recently revealed significant differences in microbiome composition between symbiotic versus aposymbiotic individuals reared in the laboratory [60]. In tropical and subtropical anemones and corals, *Symbiodinium* activity levels have been suggested as a driver of coral microbiome composition [61, 62], and the release of excess *Symbiodinium* photosynthate is thought to regulate microbiome composition in the mucus layer [63, 64]. In *A. poculata*, it is unknown if excess photosynthate is released to the mucus layer. Indeed, recent studies examining wound regeneration dynamics in *A. poculata* provide evidence that energy may be stored in the deep-bodied polyps. Central recovery, rather than perimeter recovery, was the dominant mode of polyp regeneration in *A. poculata*, demonstrating relatively high-energy reserves on a per-polyp basis [35, 36]. It is also possible that *Symbiodinium psygmophilum* photosynthesis in *A. poculata* does not constitute a major supply of organic matter for either the microbiome or the host, even during the warmest summer months. Dimond and Carrington [32] showed that heterotrophic feeding on organic particles was the main form of carbon acquisition even in symbiotic *A. poculata*, with pigmentation (symbiotic state) explaining only 23% of the variation in host growth rate. Given the high reliance of *A. poculata* on particle ingestion, microbiome members may respond to changes in host heterotrophic intake and waste nutrient availability, rather than differential levels of *Symbiodinium* growth rate, density, or pigmentation.

Given the similarity of symbiotic versus aposymbiotic *A. poculata* microbiomes and the lack of similarity between coral and seawater microbiome composition at each time point (Fig. 4), the seasonal fluctuation in *A. poculata* microbiomes is likely due to factors other than *Symbiodinium* density or changes in water column microbiomes. Such factors could include shifts in host physiology associated with the transition to quiescence in winter months and emergence from quiescence during the spring months. During cold temperatures and winter quiescence, *A. poculata* retracts its polyps and becomes unresponsive to touch [32, 33], feeds less, loses biomass, and allocates less energy to growth, regeneration, and repair [35]. It is possible that springtime emergence of the holobiont from quiescence, which includes an increase in host feeding [33] and therefore presumably changes in host energy allocation and substrate supply for microbial growth, could drive a significant re-structuring of the microbiome. In our study, microbiomes from the *A. poculata* colonies collected in the spring exhibited the highest alpha diversity (Fig. 2) and the lowest inter-individual beta diversity (Fig. 4a), indicating that in the spring, *A. poculata* harbors a diverse but predictably structured microbiome relative to communities from the other seasonal time points. In contrast, inter-individual beta diversity in seawater samples varied little between winter and spring, suggesting that the decline in beta diversity in the spring *A. poculata* microbiomes does not simply reflect a seasonal change in microbial composition in the overlying seawater. Previous studies in other systems have shown that seasonal shifts and phenological timing have co-varied with microbiome composition [65, 66] and that increased beta diversity of complex host-associated microbiomes may reflect a response to disturbance or an overall destabilization of the microbiome from one configuration, or stable state, to another. This pattern has been demonstrated in corals [54, 67] and in mammals [68, 69]. Taken together, these results raise the possibility that the microbiome of *A. poculata* shifts significantly in response to the transition from quiescence to holobiont activity in spring. This transition appears to homogenize microbiome structure across individuals, potentially reflecting a conserved functional response of the microbiome to an increase in holobiont metabolic rates, and is consistent with the recent observation of periodic microbiome succession in the tropical coral *Porites astreoides* in response to cyclical mucus shedding [70].

### Microbiome members

The seasonal shifts in *A. poculata* microbiome composition primarily involved changes in the relative abundance of common bacterial classes. This pattern is consistent with recent assertions that microbiomes respond to stress or disturbance through community-level shifts in proportional representation, rather than the disappearance or appearance of specific taxa, including pathogens [26, 67]. Notably, the wintertime *A. poculata* microbiome was enriched in Clostridiaceae, Oscillatoriaceae, and Rickettsiaceae, but contained fewer Oceanospirillaceae relative to other seasons. Similar trends have been proposed as indicators of a wide range of disturbances in tropical corals, including nutrient enrichment and temperature increases [26, 27, 67]. However, the winter populations of *A. poculata* sampled in this study did not exhibit signs of disease, and *A. poculata* has been documented to survive quiescence during extended periods of extreme low winter temperatures [32]. Thus, the *A. poculata* wintertime microbiome likely does not reflect a disease state, but potentially a general “dysbiosis” due to meta-organism quiescence or lack of host metabolic activity that serves to regulate microbiome structure (as described in [71]). It is therefore feasible that what has been observed as disease-associated microbiome signatures (i.e., the “pathobiome”; (reviewed in [72])) in tropical corals may instead be indicators of loss of regulation by the meta-organism or a lack of restriction of microbial opportunists that are not typically a significant portion of the coral’s core microbiome.

In *A. poculata*, as in coral-microbe associations in general, the proportion of detected microbial taxa that are functionally significant to the holobiont is not known. Of the nearly 200,000 OTUs detected from mucus, tissue, and skeleton of the tropical coral *Pachyseris speciosa*, only nine bacterial taxa were detected in ≥ 90% of the tested colonies [73]. Such studies suggest that the vast majority of coral microbiome-derived sequences are from taxa that exhibit varying degrees of host association, although the enzymes encoded by these taxa may contribute to a core set of biochemical pathways intrinsic to holobiont function. Among the 25 OTUs that were present across all *A. poculata* samples (Additional file 5), a single OTU was classified as belonging to the ammonia-oxidizing archaeal genus *Nitrosopumilus*, which has previously been shown to be significant to nitrogen cycling in sponges, corals, and coral reef sand and sediment [74–76]. Also present in 100% of coral samples were OTUs representing Amoebophilaceae, Pirellulaceae, and Plancomycetaceae, although the latter two families were present in very low abundance. Amoebophilaceae sequences were more abundant. This family has been found previously in corals and hypothesized to include symbionts of *Symbiodinium* or other protists in the coral holobiont [55]. The *Amoebophilus* and *Nitrosopumilus* OTUs recovered here did not change significantly in relative abundance across seasons, indicating that they may be stable, functionally significant members of the *A. poculata* microbiome. Although *Amoebophilus* sp. has been proposed as a *Symbiodinium* associate in tropical corals, in *A. poculata*, this OTU is not enriched in brown colonies. An OTU of the genus *Endozoicomonas*, which has been recovered from a wide variety of cnidarians and other invertebrates and also shown to occur in aggregates within coral tissue [15, 55], was recovered across 94% of the *A. poculata* samples. Together, the OTUs common across our coral samples may be targets for further study to better understand functional contributions of the *A. poculata* core microbiome. Recent studies suggest that microhabitats or niches within corals (i.e., the mucopolysaccharide layer, tissue, and skeleton) harbor specific microbial communities [77]. In this study, the microbiome of the entire coral was examined, with all potentially distinct microhabitats pooled together. Future research that focuses on the microbiomes of separate microhabitats within a single colony would provide useful insight into the potential stability and function of key microbiome players.

### Comparison with tropical corals

The *A. poculata* colonies in this study, taken from the northern edge of the species range [78], exhibited lower alpha diversity than has been documented in tropical corals via comparable sequencing methods. For example, Chao1 richness estimates in colonies of the Great Barrier Reef coral *Pachyseris speciosa* range from 700 to 1500 OTUs [73]. In contrast, the average Chao1 estimate in the *A. poculata* microbiomes was 471(± 118), ranging from 213 to 692. Notably, alpha diversity of cold, deep-water corals has been shown to be even lower [79]. Furthermore, within each seasonal time point, overall microbiome composition in *A. poculata* was remarkably uniform between samples, a pattern that also differs from that of tropical scleractinian coral microbiomes [80]. It is possible that emergence from quiescence may catalyze an annual “re-organization” of the *A. poculata* microbiome each spring. Drastic fluctuations in environmental parameters, including temperature and levels of nutrients or organic carbon might also drive cyclical microbiome community succession, including the spring re-organization. It is possible that such re-organization, regardless of its cause, resets the coral microbiome annually, resulting in the observed high levels of stability in the microbiome. This pattern would not be expected in tropical corals, which do not undergo a dormant phase.

## Conclusions

Here, the facultative nature of the *A. poculata-S. psygmophilum* symbiosis has enabled us to distinguish the role of season from the role of symbiosis in structuring the coral microbiome. Although seasonal shifts in *A. poculata* microbiome composition do occur, our data suggest that they are not due to fluctuation in *Symbiodinium* density. We observed a re-structuring of the *A. poculata* microbiome upon transition from winter to spring, during which time microbiome composition became more similar among host individuals. This pattern raises the hypothesis that the microbiome undergoes succession correlated with season, potentially including a shift from a destabilized microbiome in quiescent colonies during the coldest winter months, followed by a re-structuring in the spring as temperatures rise and holobiont activity resumes. At the microbial class level, *A. poculata* microbiomes are similar to those in tropical corals, being dominated, for example, by γ- and α-proteobacteria, with Cytophagia, Flavobacteriia, δ-proteobacteria, and Thaumarchaea present at lower abundances. These trends suggest potential similarities in microbial metabolic profiles and contributions to holobiont function, although this hypothesis requires further testing. Future studies should extend across the entire range of *A. poculata*, including corals from regions that experience less extreme seasonal fluctuations in temperature and nutrient/organic matter levels. Indeed, it is not yet known if quiescence occurs in southern populations of *A. poculata*. Studies in regions where *A. poculata* occurs with tropical corals (e.g., the Gulf of Mexico) will enable direct comparisons of microbiome organization drivers across diverse hosts, potentially helping to identify unifying principles of microbiome assembly. The importance of phenology in the stability of coral microbiomes, both temperate and tropical, will become increasingly important under climate change [81]. A continued increase in the severity and frequency of high-temperature periods on tropical reefs, which have otherwise not historically experienced wide temperature fluctuations, could drastically alter the ability of corals to regulate microbiome composition.

## Additional files

Additional file 1: Two-way ANOVA demonstrating that both seasonal time point and symbiotic state affect *Symbiodinium* density. (XLSX 34 kb)

Additional file 2: Distribution of *A. poculata* colonies at Ft. Wetherill, RI, surveyed in July. Symbiotic (brown), aposymbiotic (white), and mixed (hashed) colonies occur in close proximity, not stratified by depth. (TIF 677 kb)

Additional file 3: NMDS visualizations of beta diversity among coral samples from summer, fall, and winter time points only. NMDS clustering is based on the Bray–Curtis dissimilarity metrics. Ellipses represent 95% confidence intervals. ANOSIM (box within NMDS plots) also revealed significant dissimilarity between groupings of coral samples from the three seasonal time points (A), although these differences were slight. (XLSX 709 kb)

Additional file 4: DESeq2 analysis results, showing log_2_ fold-change values for genera that exhibited significant shifts in relative abundance from one season to another. Genera that were represented by five or fewer reads across all coral samples were removed from analysis. A total of 181 genera show a significant difference in at least one of the season-to-season comparisons. A positive value indicates a decrease in abundance from the first season to the next; a negative value indicates an increase in abundance from the first season to the next. (XLSX 58 kb)

Additional file 5: OTUs consistently detected in ≥ 90% of *Astrangia poculata* colonies in this study. OTUs identified to the lowest taxonomic level possible, with corresponding percentage of occurrence within the test samples from this study. (XLSX 12 kb)
