# Supplementary figures and images for: Season, but not symbiont state, drives microbiome structure in the temperate coral Astrangia poculata
Source: Microbiome. 2017 Sep 15;5:120. doi: 10.1186/s40168-017-0329-8 (PMC5603060; doi:10.1186/s40168-017-0329-8)

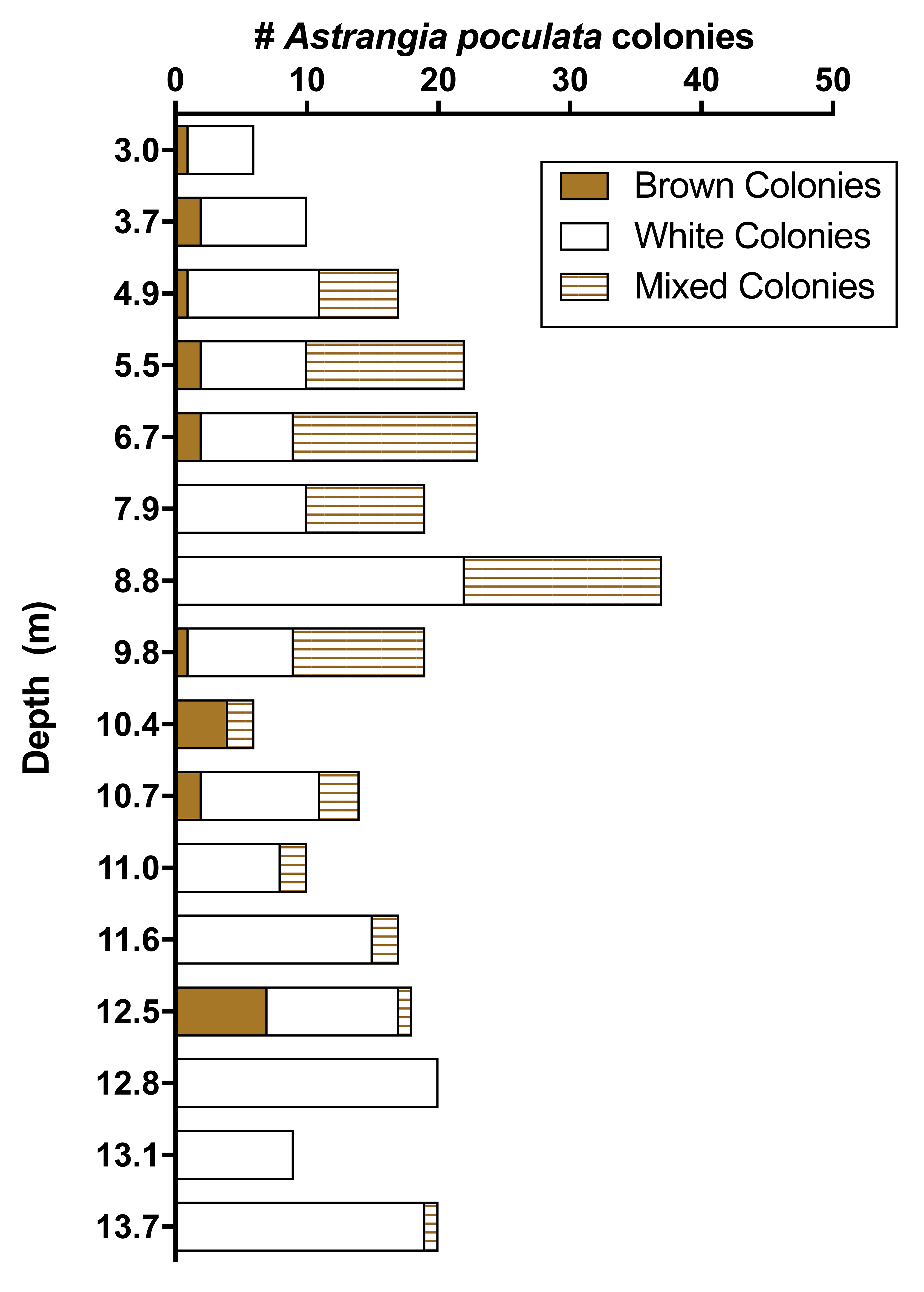

Supplement: Supplementary file 2 — Distribution of A. poculata colonies at Ft. Wetherill, RI, surveyed in July. Symbiotic (brown), aposymbiotic (white), and mixed (hashed) colonies occur in close proximity, not stratified by depth. (TIF 677 kb) [file 40168_2017_329_MOESM2_ESM.tif]

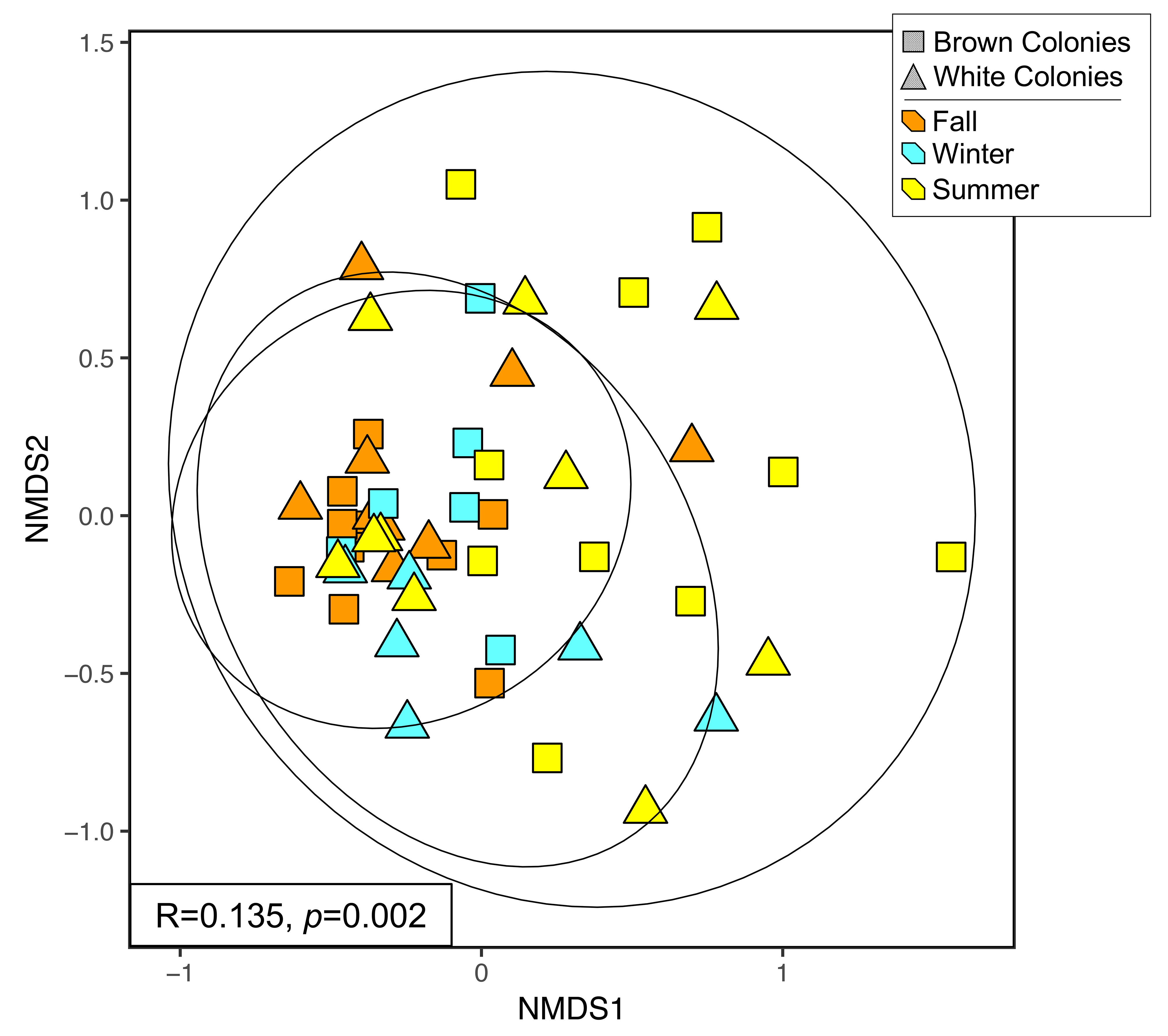

Supplement: Supplementary file 3 — NMDS visualizations of beta diversity among coral samples from summer, fall, and winter time points only. NMDS clustering is based on the Bray–Curtis dissimilarity metrics. Ellipses represent 95% confidence intervals. ANOSIM (box within NMDS plots) also revealed significant dissimilarity between groupings of coral samples from the three seasonal time points (A), although these differences were slight. (XLSX 709 kb) [file 40168_2017_329_MOESM3_ESM.tif]
